# Danger Control Programs Cause Tissue Injury and Remodeling

**DOI:** 10.3390/ijms140611319

**Published:** 2013-05-28

**Authors:** Jan H. Hagemann, Holger Haegele, Susanna Müller, Hans-Joachim Anders

**Affiliations:** 1 Nephrologisches Zentrum, Medizinische Klinik und Poliklinik IV der Ludwig-Maximilians Universität, München 80336, Germany; E-Mails: janhenrik.hagemann@med.lmu.de (J.H.H.); holger.haegele@med.uni-muenchen.de (H.H.); 2 Pathologisches Institut, Ludwig-Maximilians Universität, München 80336, Germany; E-Mail: susanna.mueller@med.uni-muenchen.de

**Keywords:** regeneration, fibrosis, coagulation, stem cells, inflammation, acute kidney injury, chronic kidney disease, healing, repair

## Abstract

Are there common pathways underlying the broad spectrum of tissue pathologies that develop upon injuries and from subsequent tissue remodeling? Here, we explain the pathophysiological impact of a set of evolutionary conserved danger control programs for tissue pathology. These programs date back to the survival benefits of the first multicellular organisms upon traumatic injuries by launching a series of danger control responses, *i.e.*, 1. Haemostasis, or clotting to control bleeding; 2. Host defense, to control pathogen entry and spreading; 3. Re-epithelialisation, to recover barrier functions; and 4. Mesenchymal, to repair to regain tissue stability. Taking kidney pathology as an example, we discuss how clotting, inflammation, epithelial healing, and fibrosis/sclerosis determine the spectrum of kidney pathology, especially when they are insufficiently activated or present in an overshooting and deregulated manner. Understanding the evolutionary benefits of these response programs may refine the search for novel therapeutic targets to limit organ dysfunction in acute injuries and in progressive chronic tissue remodeling.

## 1. Introduction

Most acute and chronic disorders involve a combination of direct and indirect tissue injuries, *i.e.*, the damage caused by the injurious trigger, and that caused by a series of different danger response programs, *i.e.*, clotting, inflammation, epithelial regeneration, and mesenchymal repair. These processes predominate in a serial manner during acute disorders, with some overlap. However, in chronic non-communicable diseases this overlap turns into a concomitant persistence of some of these programs, e.g., inflammation and healing, which often leads to significant parenchymal atrophy and fibrosis. In this review we use kidney pathology as an example for the spectrum of pathological manifestations that derive from these danger response programs and explain why evolution’s risk-benefit assessment conserved them across species over time. Better understanding of the origin of tissue pathologies should be instrumental to better define target pathways for therapeutic interventions.

## 2. Injuries Trigger a Series of Host Response Programs

Wounding requires immediate actions to control the dangers that come with the injury followed either by regeneration or repair, a concept that applies to plants and animals [1,2]. Focal wounding requires local danger control, e.g., of pathogen entry, to prevent systemic consequences such as fatal sepsis. Hence, such survival benefits from local danger control, outweighs any risk of focal collateral injury or tissue remodeling that comes with these danger responses. What remains problematic are systemic triggers of danger responses, such as toxic (drugs), hemodynamic (shock or arterial hypertension), or metabolic alterations (diabetes, hyperlipidemia) because these induce responses that affect multiple organ systems and organ compartments, which implies that any collateral damage threatens entire organs, if not the whole body. In the following paragraphs we will introduce the four major danger response programs along wound healing after acute skin injury [3–5].

### 2.1. Clotting Addresses the Risk of Potentially Fatal Bleeding

Skin injury causes bleeding, which implies the risk of dying from hemorrhagic shock. Clotting addresses this type of danger within minutes by haemolymph aggregation in arthropods, and by a more sophisticated interplay of injured endothelial cells, coagulation factors, and platelets in vertebrates [6–9]. Overshooting clotting causes tissue ischemia via intravascular coagulation or thromboembolism.

### 2.2. Inflammation Addresses the Risk of Fatal Sepsis

The balance between microbe virulence and host defense has gained considerable complexity along the evolution of monocellular organisms [10–13]. Outer surface wounding allows pathogen entry and may lead to fatal sepsis, if not immediately addressed by local inflammation to control pathogen spreading [14,15]. Extrinsic pathogen-associated molecular patterns (PAMPs) and intrinsic damage-associated molecular patterns (DAMPs) act as alarmins that activate the same innate immunity pattern recognition receptors in infectious or sterile inflammations [16–21]. Inflammation is already activated by clotting, a process referred to as immunothrombosis [6,22–24], as platelet aggregates release chemokines that trigger the recruitment of neutrophils [6,25,26]. Overshooting local inflammation causes unnecessary immunopathology and loss of parenchyma, e.g., in pyoderma gangraenosum [27,28]. Overshooting systemic inflammation contributes to the early phase of sepsis, while insufficient systemic inflammation accounts for lethality in the late phase of sepsis [14,29].

### 2.3. Epithelial Regeneration Restores Barrier Functions

Non-sterile barrier defects require rapid regeneration of the barrier to limit pathogen entry [5,30]. Epithelial barriers have also other important functions, such as nutrient absorbtion (gut), gas transfer (lungs), or solute secretion/re-absorption (kidney), which require rapid restoration upon injury, e.g., by signals that trigger re-epitheliasation from wound borders [2,5,31]. Components of the coagulation cascade are the first mediators inside a wound that elicit mitogenic effects on the surviving epithelial cells [2,7,25,31,32]. Inflammatory mediators with mitogenic properties, such as epithelial growth factors, hepatocyte growth factor, IL-6, IL-17, fractalkine, CXCL10, and IL-22, as well as certain miRNAs, stimulate epithelial repair [31,33–40]. Local progenitor cells that are committed to the specific epithelial lineage phenotype contribute to re-epithelialisation [30,40–42]. Insufficient re-epitheliasation creates chronic wounds, which implies a risk of infections, while overshooting or uncoordinated re-epitheliasation, can also cause, problematically, hyperplastic lesions [2,43].

### 2.4. Mesenchymal Repair Restores Tissue Stability

Insufficient re-epithelialisation, loss of tissue parenchyma, or injury to mesenchymal tissues activates the wound healing program of mesenchymal repair. This process is needed to stabilize the organ’s shape and structure. Insufficient epithelial repair directly stimulates mesenchymal healing, as epithelial-mesenchymal transition (EMT) of epithelial cells, and their arrest in the G2/M phase of the cell cycle induce the secretion of the pro-fibrotic cytokine TGF-β [44]. The associated accumulation of collagen-producing cells [45,46] relates to an influx of bone marrow-derived fibrocytes [47,48], a mesenchymal transition of also pericytes and endothelial cells [48,49], as well as the proliferation of resident fibroblasts that transform to myofibroblasts [49]. The accumulation of extracellular matrix (referred to as fibrosis) stiffens the tissue (referred to as sclerosis). Insufficient mesenchymal healing destabilizes tissues while overshooting mesenchymal healing produces fibrotic lesions such as keloid, and diffuse fibrotic disorders such as scleroderma.

**Figure 1:**
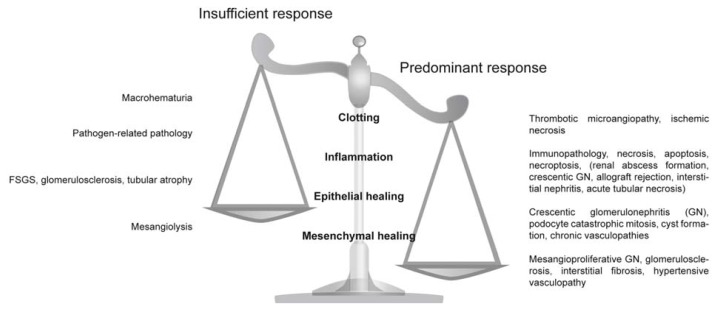
Insufficient and predominant danger response programs determine kidney pathology. Distinct entities of clinical syndromes or kidney injury patterns are consequences of insufficient or overshooting danger control programs.

In the following we discuss how these ancient danger control programs account for the spectrum of organ abnormalities known from pathology textbooks. Based on our own experience we focus to kidney pathology. We describe in more detail how common histomorphological abnormalities and disease entities develop, either from insufficient, or overshooting danger control programs (Figure 1).

## 3. Clotting

### 3.1. Overshooting Clotting in the Kidney

**Figure 2:**
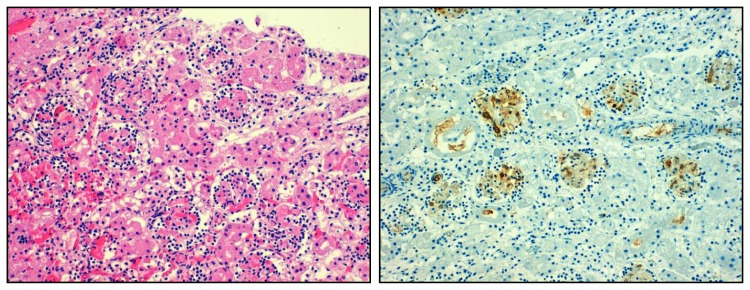
Overshooting clotting in thrombotic microangiopathy. Local activation microvascular endothelial cells and lack of distinct inhibitory factors can lead to thrombotic microangiopathy which is characterized by microthrombi obstructing arteriolar and glomerular vessels, a lesion associated with significant inflammatory cell infiltrates (**left**). Fibrin immunostaining displayes clot formation within glomeruli (**right**). Original magnification 200×.

Thrombotic microangiopathies that affect the kidney result from an abnormal activation of microvessel endothelial cells, and subsequent activation of platelets and plasmatic coagulation (Figure 2) [50,51]. Microvascular clotting causes tissue ischemia and necrosis. In crescentic glomerulonephritis, vascular necrosis leads to perforations, ruptures in the glomerular basement membrane (GBM), plasma leakage, and bleeding [52]. Glomerular bleeding manifests clinically as hematuria, a process that should activate clotting inside the glomerulus. In fact, fibrin deposition is usually seen in areas of loop necrosis in crescentic glomerulonephritis, in turn, used as a diagnostic marker to identify necrotizing glomerulonephritis [53,54]. In Alport nephropathy, a genetic GBM abnormality not associated with intense inflammation, progressive intrinsic degradation and disintegration of the GBM, generates similar GBM perforations and ruptures that are associated with hematuria, fibrin deposits, and plasma leakage [43]. As part of the clot formation, activated platelets release pro-inflammatory and mitogenic factors, which activate subsequent inflammation as well as and epithelial regeneration [7,25,26,31,55]. The link of clotting and inflammation, referred to as immmunothrombosis, is well defined in the field of immunology and currently understood as a mechanism to limit pathogen spreading from the entry site [6,56–59]. This evolutionary conserved process certainly contributes to atherosclerosis and -thrombosis, and it is reasonable to assume that this link is as important in other non-communicable disorders.

### 3.2. Insufficient Clotting in the Kidney

IgA nephropathy, and several other renal disorders, may present with intermittent macrohematuric episodes, implicating that vascular sealing by clotting is insufficient [60]. Urokinase expression further down the urinary tract elicits fibrinolytic activity that may maintain the bleeding [61].

## 4. Inflammation

### 4.1. Overshooting Inflammation in the Kidney

Resident and infiltrating mononuclear phagocytes express the entire spectrum of innate pattern recognition receptors that translate danger recognition into a rapid inflammatory response [62,63]. In contrast, renal parenchymal cells, *i.e.*, mesangial cells, endothelial cells, podocytes, tubular cells, and fibroblasts express only a limited spectrum of such receptors [64,65]. For example, they lack some of the endosomal nucleic acid-specific Toll-like receptors, and do not produce IL-1beta upon activation of the NLRP3 inflammasome, even though they easily get activated by bacterial endotoxin or other PAMPs and DAMPs [66,67]. This mechanism can explain how systemic or extrarenal infections can cause flares of pre-existing and smoldering forms of renal inflammation, e.g., chronic glomerulonephritis or renal vasculitis. We had addressed this concept by transiently injecting agonists to several pattern recognition receptors in mice with experimental immune complex glomerulonephritis, which then triggered the intrarenal production of cytokines, type I interferons, which increases inflammation and tissue damage [65,68–77]. PAMP-mediated renal inflammation induces the loss of renal parenchymal cells, especially podocytes [78,79], because these cannot be easily regenerated [80]. For example, mice with Alport nephropathy transiently exposed to bacterial CpG-DNA, display a transient activation of resident dendritic cells and infiltrating Ly6Chigh+ macrophages that produce pro-inflammatory mediators such as TNF, which triggered podocyte loss and thereby accelerated proteinuria and glomerular scarring [78].

In lupus nephritis, endogenous nucleic acids drive immunity in a similar manner [81,82]. Nuclear particles that contain immunostimulatory endogenous RNA and/or DNA activate antigen-presenting dendritic cells, macrophages, and B cells, to mature and to release numerous pro-inflammatory mediators including type I interferon [82–84]. The latter sets off a coordinated antiviral immune response explaining the similarities between the clinical manifestations of viral infections and systemic lupus [85]. This process also occurs inside the kidney, as documented by the antiviral gene expression signature in renal biopsies [86,87]. For example, mesangial cells and glomerular endothelial cells use their cytosolic nucleic acid sensors to translate nucleic acid recognition into the release of type I interferons, which contribute to renal inflammation and tissue damage [88–93].

Tissue necrosis is associated with a release of endogenous ligands to pattern recognition receptors [64,94,95]. For example, postischemic tubular cell necrosis releases HMGB1 and histones that ligate TLR2 and TLR4, which trigger an acute intrarenal inflammatory response that determines the extent of AKI [66,95–99]. In addition, Tamm-Horsfall protein/uromodulin, a kidney-specific protein exclusively expressed within the distal tubule, acts as a TLR4 and NLRP3 agonists when tubular injury allows its leakage into the renal interstitium [100,101]. TLR signaling occurs in renal parenchymal cells [64] and is tightly regulated in the intrarenal network of dendritic cells by the constitutive and induced expression of several inhibitory molecules that are absent or dysfunctional in tubular epithelial cells [102–108]. In contrast, the NLRP3 inflammasome enhances tubulointerstitial but not to glomerular inflammation [19,109–111], because activated glomerular cells do not induce pro-IL-1β [110].

**Figure 3:**
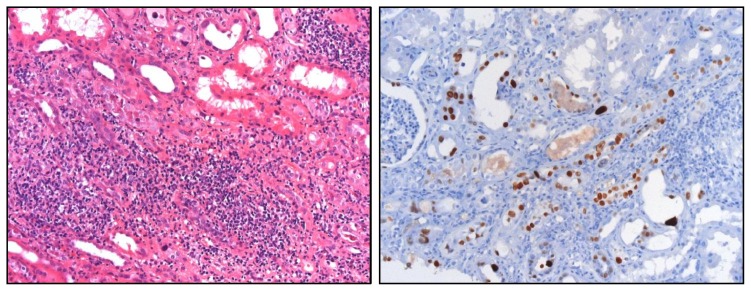
Interstitial nephritis in polyoma (BK) virus nephropathy. Local activation of inflammation can destroy renal parenchyma and cause acute kidney injury. In this example of BK virus reactivation in a kidney allograft was proven by immunostaining for BK viral protein with positivity in affected tubular epithelial cells (**right**). Viral replication activates antiviral immunity and immunopathology as evidenced by the dense leukocyte infiltrates in the renal interstitium (**left**). Original magnification 200×.

Triggered by the local release of chemokines, various subsets of leukocytes sequentially recruit into the kidney [112–120]. Macrophages, T cells, and B cells polarize into functionally distinct subsets that differently affect renal pathology [62,114,121–125]. PAMPs and DAMPs turn non-activated intrarenal and circulating mononuclear phagocytes into cells that promote immunopathology [126–128]. Blocking the CC-chemokine CCL2, or its receptor CCR2, prevents the recruitment and expansion of such classically-activated macrophages, and thereby reduces renal immunopathology in glomeruli and the tubulointerstitium, but it does not affect alternatively-activated macrophages [129–137]. Together, the kidney is mostly affected by renal inflammation that is triggered by extrarenal infections that release immunostimulatory PAMPs into the circulation, or by intrarenal release of DAMPs that promote a sterile inflammatory response [138,139]. These immunostimulatory molecules promote unnecessary (collateral) damage to renal cells (Figure 3).

Thus, suppressing renal inflammation appears as an important strategy to preserve renal tissue, especially those epithelial cells that cannot be easily regenerated. Anti-inflammatory drugs that do not elicit systemic immunosuppressive effects hold new promise for that [140].

### 4.2. Insufficient Inflammation in the Kidney

While TLR-mediated renal inflammation contributes to inappropriate renal immunopathology in sterile nephropathies, it remains an important element of pathogen control in renal infections [66,141]. For example, some degree of innate immune activation is needed to keep BK virus in check to avoid viral replication and BK virus infection of the allograft [142,143]. The enigmatic importance of renal inflammation as part of the local host defense becomes evident also during bacterial pyelonephritis [144]. Intrarenal host defense is needed to limit pathogen growth and spreading to systemic infections, which happens in TLR4-mutant mice inoculated with uropathogenic *E. coli*. While these mice are protected from renal abscess formation [145], this occurs at the price of insufficient pathogen control at the entry site and could cause fatal gram negative sepsis [14]. In addition, TLR2 is required for the recognition of leptospiral outer membrane proteins in proximal tubular epithelial cells [146].

## 5. Epithelial Regeneration

Epithelial cells determine most of the kidney’s functions, in the glomerular compartment (filtration barrier) as well as the tubular compartment of the kidney (reabsorbtion and secretion). Transient and short-term injuries to the tubules are usually followed by sufficient epithelial regeneration that rapidly restores renal function [147,148]. Numerous growth factors such as HGF, PDFG, EGF, and BMP-7 drive the repair of the epithelial monolayers after injury [7,25,31,149]. In addition, the cell cycle regulator murine double minute (MDM)-2 assures cell cycle entry of surviving tubular cells by inhibiting p53-dependent cell cycle arrest [150], in addition to its role in NF-κB signaling [150,151] Epithelial regeneration becomes effective only after the resolution of inflammation [2,5,152]. Switching the phenotype of macrophages from a pro-inflammatory (M1) toward an anti-inflammatory (M2) is important in this process [122,125,153–156]. This process is associated with the release of additional growth factors that drive epithelial recovery in the kidney, including CSF-1 that enhances M2 macrophage accumulation [137,152,155–159]. However, epithelial regeneration needs to be tightly balanced to avoid renal pathology [80].

### 5.1. Overshooting Epithelial Regeneration in the Kidney

**Figure 4:**
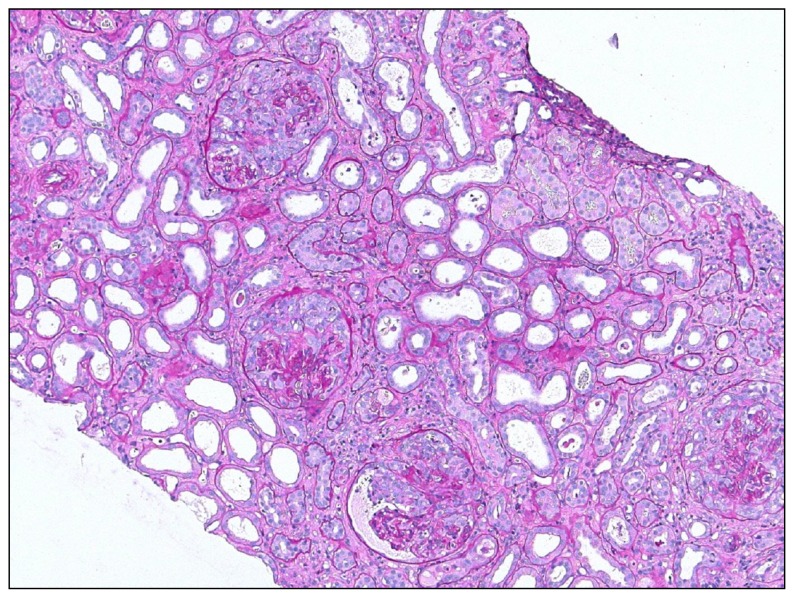
Overshooting epithelial regeneration in crescentic glomerulonephritis. Massive and uncoordinated proliferation of parietal epithelial cells leads to crescent formation in Bowman’s space, e.g., in necrotizing renal vasculitis. Original magnification 100×.

When epithelial (progenitor) cells get heavily activated in the absence of the necessary signals for differentiation, overshooting and maladaptive epithelial repair results in additional renal pathology [80,160,161]. In rapid progressive glomerulonephritis glomerular vascular necrosis, and subsequent activation of the coagulation cascade, drive intense intraglomerular inflammation [162–164]. Both, epithelial injury and inflammation induce the proliferation of parietal epithelial cells without their differentiation into podocytes, which would be required for podocyte regeneration [43,161–163]. The resulting parietal epithelial cell hyperplasia generates the initial step in glomerular crescent formation and nephron loss (Figure 4). This overshooting epithelial hyperplasia does not necessarily require inflammation as a trigger. In *Col4A3*-deficient mice disruption of glomerular capillaries was sufficient to trigger parietal epithelial cell hyperplasia [43]. Plasma leakage seemed to be the mitogenic stimulus for these epithelial cells that normally reside, devoid of serum contact, in the urinary space [165,166].

Epithelial hyperplasia in the tubular compartment is less obvious. The tubular progenitor cells are located at the junction of glomeruli and proximal tubules, while single progenitor cells are scattered in the proximal and distal tubules of the cortex [167,168]. Chevalier *et al.*, have recently demonstrated that the phenomenon of atubular glomeruli originates from an obstruction of the tubular lumen by epithelial cells [169,170], a process that contributes to tip lesions in focal-segmental glomerulosclerosis and diabetic nephropathy [171,172].

### 5.2. Insufficient Epithelial Regeneration in the Kidney

**Figure 5:**
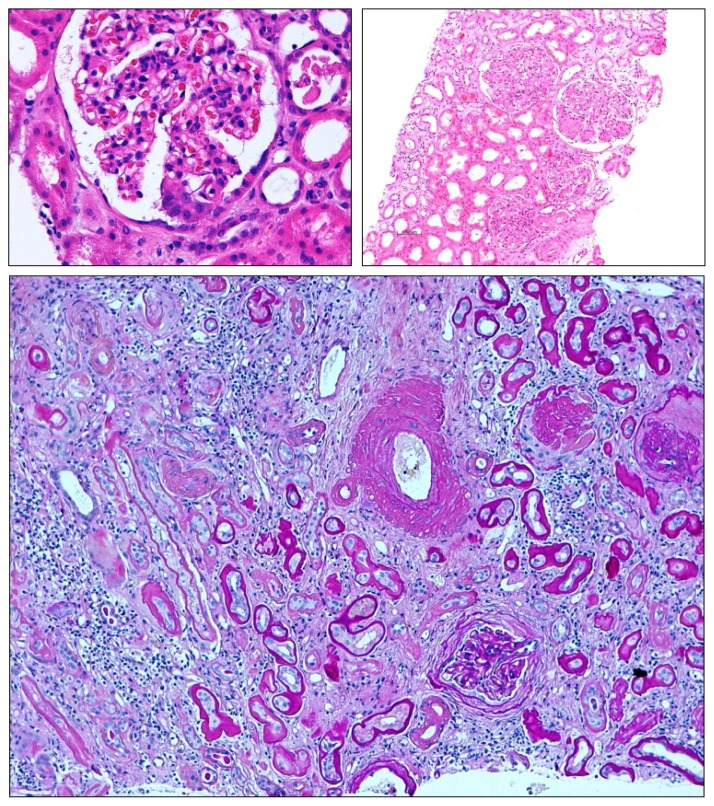
Insufficient epithelial repair results in mesenchymal repair. Loss of glomerular epithelial cells (podocytes) cannot be easily repaired in adults leading to focal-segmental glomerulosclerosis (**upper left**). Global glomerulosclerosis (**upper right**) results from the progression of focal-segmental lesions or as a consequence of diffuse and persistent disease mechanisms such as diabetes, hypertension, or immune complex glomerulonephritis. Here also, mesangial cells contribute to the sclerosis by excess production of mesangial matrix. In chronic kidney disease insufficient tubular regeneration results in tubular atrophy, which is usually associated concomitant tubulointerstitial fibrosis. These complex lesions make it difficult to appreciate the individual contributing danger response programs. Magnification 100×–400×.

Insufficient glomerular epithelial (podocyte) regeneration is the predominant cause for chronic kidney disease (CKD) and for progression to end stage renal disease (ESRD) (Figure 5). The particular interaction of differentiated podocytes with each other, and with the GBM that maintains the glomerular filtration barrier remains a major obstacle for rapid repair [173–176]. This is because podocytes involve all their cytoskeleton to maintain the secondary foot processes and the slit diaphragm, which is not compatible with reorganizing the cytoskeleton to form the mitotic spindle during mitosis [177]. Thus, when podocytes enter the S phase of the cell cycle to undergo hypertrophy, cell cycle arrest at the G1 and G2/M restriction points are needed to prevent the podocyte to undergo mitosis or, otherwise, podocytes will subsequently detach and die, *i.e.*, mitotic catastrophe [177–180]. There has been a controversial debate whether bone marrow-derived progenitors are able to replace lost podocytes [181–183], but meanwhile it has been demonstrated that podocytes originate from local epithelial progenitors at the urinary pole of the glomerulus that can migrate to the vascular pole and differentiate into terminally differentiated podocytes on the glomerular tuft [178,184,185]. This process contributes to renal development and podocyte expansion during kidney growth in early childhood [101] but its capacity to replace injured podocytes seems to be limited in adults [184,186]. The signaling pathways that regulate podocyte renewal from parietal epithelial cells remain to be clarified. Notch and Wnt signaling, EGF and SDF-1/CXCL12 seem to have a role [164,178,187,188]. Specific epigenetic imprinting at histone H3K9, H3K23 (acetylation), H3K4 (dimethylation), and H3K4 phosphorylation at serine 10, which alters gene expression and cell growth, are associated with incomplete podocyte recovery [189,190].

The persistence of classically-activated mononuclear phagocytes or repetitive/persistent triggers of kidney injury also impair epithelial repair in the tubulointerstitial compartment. CSF-1 is a mediator of this process [159]. In addition, severe kidney injury may eradicate the tubular progenitor cells that have a higher capacity to survive stress [191]. Bone marrow stem cells do not directly replace tubular cells by differentiation, but provide paracrine support to the regeneratory capacity of local progenitor cells and other surviving epithelial cells [154,168,192–194]. An insufficient regeneration of injured tubular epithelial cells will lead to tubular atrophy and nephron loss, a typical characteristic of progressive CKD (Figure 5).

Together, a coordinated epithelial regeneration is needed upon injury, which first requires vascular sealing and the resolution of inflammation. Insufficient epithelial regeneration leads to atrophy and mesenchymal repair, *i.e.*, tubular atrophy and glomerulosclerosis. Overshooting epithelial repair, *i.e.*, hyperplasia, is another form of renal pathology, e.g., the glomerular crescent. Finding ways to enhance a coordinated proliferation and differentiation of surviving epithelial cells remains a challenge for the future.

## 6. Mesenchymal Repair

Tissue healing is not limited to epithelia but also includes recovery of vasculature, tendons, fasciae, bones, and muscles, all being mesenchymal structures that contribute to function, not only of the musculoskeletal system but also to that of solid organs. For example, mesenchymal repair contributes to mechanical stabilization of organs like the lung, the heart, and the kidney, which is why epithelial growth factors are secreted together with growth factors that stimulate mesenchymal repair, e.g., by increasing the secretion of extracellular matrix components [31]. In skin wounds, dermal fibroblasts get activated to transform into myofibroblasts that produce large amounts of type I collagen, which promotes wound contraction as a way to reduce wound size for more rapid re-epithelisation [2]. Irreversible tissue losses such as in ruptured ligaments or burned skin get filled by fibrous tissue to regain tissue stability [2]. These benefits of mesenchymal repair explain why this response program was positively selected and maintained along evolution. Solid organs like the kidney, however, often suffer from global scarring and progressive fibrosis because of the diffuse nature of the metabolic, hemodynamic, and toxic injuries that commonly affect the kidney (Figure 5). [49]. That is why it is not at all clear whether diffuse renal fibrosis is an overshooting, or simply a diffuse response. For example, interstitial fibrosis in focal-segmental glomerulosclerosis (FSGS) starts in a focal manner around those single nephrons that succumb to scarring [173]. The diffuse appearance of interstitial fibrosis only develops once many nephrons undergo the same process. The mesenchymal “repair” of each dying nephron then leads to confluent fibrotic lesions that give the impression that it is the fibrosis that accounts for renal dysfunction [46,49,173,195]. The extracellular matrix mainly replaces lost renal epithelia. As such, reducing interstitial fibrosis may result in even smaller kidneys, if not being accompanied by sufficient generation of new nephrons, a process which fish can do, but mammals cannot [196]. For the sake of didactic clarity, we will continue to use the term “overshooting” mesenchymal repair for the discussion of fibrosis as a danger response program that accounts for renal pathology.

### 6.1. Insufficient Mesenchymal Repair in the Kidney

Insufficient mesangial repair is a hardly known phenomenon in kidney pathology, or at least poorly defined. Mesangiolysis can be considered as a lesion of incomplete mesenchymal repair, but mesangiolysis is rarely described in renal biopsies, as it is mostly a transient phenomenon followed by rapid, (and often overshooting) mesangial cell recovery [197,198]. Mesangiolysis often results from massive renal complement activation, as in C3 glomerulopathies, atypical hemolytic uremic syndrome, or immune complex glomerulonephritis [199].

### 6.2. Overshooting Mesenchymal Repair in the Kidney

Mesangial repair after inflammatory injury can occur from three sources: surviving mesangial cells [198], from the extraglomerular mesangium [200], and from the bone marrow [201]. Mesangial injury rarely occurs in an isolated manner, but complicates diseases that are associated with extensive complement activation such as hemolytic uremic syndrome. The rat anti-Thy1.1 model is frequently used to study the mechanisms of mesangial repair upon mesangiolysis. Hugo *et al.*, first reported that the hyperproliferative response upon mesangiolysis originates from surviving mesangial cells or local progenitor cells that reside in the extraglomerular mesangium [200]. Mesangial hyperproliferation also contributes to a histopathological lesion named “membrano-proliferative glomerulonephritis” where hereditary, or acquired forms, of extensive complement activation within the mesangium lead to a persistent expansion of mesangial cells that even extend into the space between the GBM and the endothelial cells [202]. The mesangial matrix produced along these mesangial cell extensions stains positive with silver, which then gives glomerular capillaries a splitted GBM appearance on light microscopy [202].

In the glomerulus this causal relationship becomes clear, as podocyte renewal from local progenitors is mostly insufficient, especially in proteinuric disorders of the adult [80,203]. In FSGS, parietal epithelial cells rather produce extracellular matrix, and generate segmental sclerotic lesions, than regenerating lost podocytes [204]. This focal synechia has still the potential to stabilize the focal loss of podocytes as in some forms of secondary FSGS [171]. However, beyond a certain amount of lost podocytes, the scarring process acquires its own dynamic and progresses to global glomerulosclerosis [175], because hyperfiltration of the remaining glomerulus adds more hemodynamic stress on the surviving podocytes [173,205]. The mesenchymal transition of parietal epithelial cells further contributes to fibrocellular crescent formation, implying an irreversible loss of the entire nephron [206]. In this way, parietal epithelial cells directly contribute, not only to epithelial, but also to mesenchymal repair [166].

The process of epithelial-mesenchymal transition of surviving epithelial cells that cannot rapidly regenerate epithelial injuries has attracted a lot of attention [46,207–209]. It is based on the observation that epithelial cells of mesenchymal origin, like the renal epithelia, re-express mesenchymal markers upon injury *in vitro* and *in vivo* [46]. This has led to the assumption that such cells could leave tubular compartment and migrate into the renal interstitium where they may fuel into the heterogeneous pool of fibroblasts and contribute extracellular matrix production and fibrotic lesions [46,49]. The significance of this phenomenon for human kidney disease remains under debate because clear evidence for tubular cells leaving the tubular compartment *in vivo* is still lacking [46,207–209]. In the glomerulus, however, parietal epithelial cells that do not adequately differentiate into podocytes clearly undergo this mesenchymal transition and cause scarring, as there is no need for them to leave their home compartment (Figure 5) [43,206].

The concept that renal interstitial fibrosis accounts for renal dysfunction originates from the close association of the extent of renal fibrosis with poor outcomes of primary glomerular disorders [210], but functional studies do not always support this causal relationship [49,196]. It is of note that the driving factor of interstitial fibrosis seems to be epithelial injury and insufficient epithelial repair [211], e.g., when proliferating epithelial cells get arrested in the G2/M phase and start to produce tumor growth factor-beta [44]. This process is also triggered by aristocholic acid [44,212], the nephrotoxic element of Chinese herb nephropathy [213]. Bone marrow progenitor cells and leukocytes enhance the process of renal fibrosis, as evidenced by experimental interventions that block leukocyte recruitment and can prevent interstitial fibrosis either as a direct or an indirect effect [112,134,214–219]. For example, inhibition, genetic deletion, or depletion of alternatively-activated (M2) macrophages protects from renal fibrosis [122,155,156,220–227]. Fibrocytes are a particular type of Ly6G+ collagen-producing cell that originate from myeloid precursors in the bone marrow and that recruit to sites of chronic kidney injury [228,229]. Ly6G+ fibrocytes specifically recruit via CCL21-CCR7 and not via CCL2-CCR2 like pro-inflammatory macrophages, but once they reach the kidney they contribute to local collagen deposition and interstitial fibrosis [230,231].

Vascular reconstruction is another element of mesenchymal healing [232]. Pericytes stabilize microvessels not only during homeostasis, but also during microvessel recovery, a process mediated by TIMP3 and ADAMTS1 [233]. Their capacity to produce collagen adds pericytes to the list of cells that contribute to renal interstitial fibrosis and sclerosis [234].

Together, mesenchymal repair is needed to stabilize and rebuild tissues after injury, especially after loss of parenchymal tissue. Insufficient scarring is rarely a problem in the kidney. In contrast, scarring within the glomerulus, usually upon dysregulated epithelial repair like in podocyte loss, or crescent formation, is the greatest concern as FSGS and fibrocellular crescents both eventually lead to loss of the entire nephron (Figure 5). Within the interstitial compartment fibroblast- and pericyte-derived extracellular matrix fills the gaps left by dying nephrons and, this way, stabilizes the remaining nephrons. This process, however, further contributes to vascular rarefication and renal ischemia and is thought to further promote the progression of kidney disease. Hence, an otherwise beneficial wound healing response turns into a maladaptive process that promotes organ failure, mainly because of the diffuse nature of most kidney diseases.

## 7. Summary

Clotting, inflammation, epithelial, and mesenchymal healing represent ancient danger response mechanisms that were positively selected throughout evolution for their benefits on host survival upon focal injury. In focal injuries the associated collateral damages may be acceptable. In contrast, in diffuse injuries, as they usually affect the kidney, these danger response programs often turn into maladaptive pathomechanisms that account for organ failure. Research efforts can benefit from dissecting these individual danger response programs, and from studying their regulatory interactions. From a therapeutic perspective, inhibiting the unnecessary inflammatory response in renal sterile inflammation and stimulating a coordinated epithelial repair should be the most promising strategies to avoid kidney pathology and disease progression.

## References

[1] Schilmiller A.L., Howe G.A. (2005). Systemic signaling in the wound response. Curr. Opin. Plant Biol.

[2] Gurtner G.C., Werner S., Barrandon Y., Longaker M.T. (2008). Wound repair and regeneration. Nature.

[3] Singer A.J., Clark R.A. (1999). Cutaneous wound healing. N. Engl. J. Med.

[4] Clark R.A. (1985). Cutaneous tissue repair: Basic biologic considerations. I. J. Am. Acad. Dermatol.

[5] Martin P. (1997). Wound healing—Aiming for perfect skin regeneration. Science.

[6] Engelmann B., Massberg S. (2013). Thrombosis as an intravascular effector of innate immunity. Nat. Rev. Immunol.

[7] Nurden A.T. (2011). Platelets, inflammation and tissue regeneration. Thromb. Haemost.

[8] Furie B., Furie B.C. (2008). Mechanisms of thrombus formation. N. Engl. J. Med.

[9] Aird W.C. (2003). The role of the endothelium in severe sepsis and multiple organ dysfunction syndrome. Blood.

[10] Medzhitov R. (2008). Origin and physiological roles of inflammation. Nature.

[11] Messier-Solek C., Buckley K.M., Rast J.P. (2010). Highly diversified innate receptor systems and new forms of animal immunity. Semin. Immunol.

[12] Gauthier M.E., Du Pasquier L., Degnan B.M. (2010). The genome of the sponge Amphimedon queenslandica provides new perspectives into the origin of Toll-like and interleukin 1 receptor pathways. Evol. Dev.

[13] Wiens M., Korzhev M., Perovic-Ottstadt S., Luthringer B., Brandt D., Klein S., Muller W.E. (2007). Toll-like receptors are part of the innate immune defense system of sponges (demospongiae: Porifera). Mol. Biol. Evol.

[14] Stearns-Kurosawa D.J., Osuchowski M.F., Valentine C., Kurosawa S., Remick D.G. (2011). The pathogenesis of sepsis. Annu. Rev. Pathol.

[15] Hickey M.J., Kubes P. (2009). Intravascular immunity: The host-pathogen encounter in blood vessels. Nat. Rev. Immunol.

[16] Chan J.K., Roth J., Oppenheim J.J., Tracey K.J., Vogl T., Feldmann M., Horwood N., Nanchahal J. (2012). Alarmins: awaiting a clinical response. J. Clin. Invest.

[17] Rock K.L., Latz E., Ontiveros F., Kono H. (2010). The sterile inflammatory response. Annu. Rev. Immunol.

[18] Anders H.J. (2010). Toll-like receptors and danger signaling in kidney injury. J. Am. Soc. Nephrol.

[19] Mulay S.R., Kulkarni O.P., Rupanagudi K.V., Migliorini A., Darisipudi M.N., Vilaysane A., Muruve D., Shi Y., Munro F., Liapis H. (2013). Calcium oxalate crystals induce renal inflammation by NLRP3-mediated IL-1beta secretion. J. Clin. Invest.

[20] Kannemeier C., Shibamiya A., Nakazawa F., Trusheim H., Ruppert C., Markart P., Song Y., Tzima E., Kennerknecht E., Niepmann M. (2007). Extracellular RNA constitutes a natural procoagulant cofactor in blood coagulation. Proc. Natl. Acad. Sci. USA.

[21] Semeraro F., Ammollo C.T., Morrissey J.H., Dale G.L., Friese P., Esmon N.L., Esmon C.T. (2011). Extracellular histones promote thrombin generation through platelet-dependent mechanisms: Involvement of platelet TLR2 and TLR4. Blood.

[22] Delvaeye M., Conway E.M. (2009). Coagulation and innate immune responses: Can we view them separately?. Blood.

[23] Niessen F., Schaffner F., Furlan-Freguia C., Pawlinski R., Bhattacharjee G., Chun J., Derian C.K., Andrade-Gordon P., Rosen H., Ruf W. (2008). Dendritic cell PAR1-S1P3 signalling couples coagulation and inflammation. Nature.

[24] Van der Poll T., de Boer J.D., Levi M. (2011). The effect of inflammation on coagulation and *vice versa*. Curr. Opin. Infect. Dis.

[25] Semple J.W., Italiano J.E., Freedman J. (2011). Platelets and the immune continuum. Nat. Rev. Immunol..

[26] Palabrica T., Lobb R., Furie B.C., Aronovitz M., Benjamin C., Hsu Y.M., Sajer S.A., Furie B. (1992). Leukocyte accumulation promoting fibrin deposition is mediated *in vivo* by P-selectin on adherent platelets. Nature.

[27] Ahronowitz I., Harp J., Shinkai K. (2012). Etiology and management of pyoderma gangrenosum: A comprehensive review. Am. J. Clin. Dermatol.

[28] Bonventre J.V., Zuk A. (2004). Ischemic acute renal failure: An inflammatory disease?. Kidney Int.

[29] Hotchkiss R.S., Coopersmith C.M., McDunn J.E., Ferguson T.A. (2009). The sepsis seesaw: Tilting toward immunosuppression. Nat. Med.

[30] Romagnani P. (2010). From proteus to prometheus: Learning from fish to modulate regeneration. J. Am. Soc. Nephrol.

[31] Werner S., Grose R. (2003). Regulation of wound healing by growth factors and cytokines. Physiol. Rev.

[32] Sopova K., Tatsidou P., Stellos K. (2012). Platelets and platelet interaction with progenitor cells in vascular homeostasis and inflammation. Curr. Vasc. Pharmacol.

[33] Braun R.K., Ferrick C., Neubauer P., Sjoding M., Sterner-Kock A., Kock M., Putney L., Ferrick D.A., Hyde D.M., Love R.B. (2008). IL-17 producing gammadelta T cells are required for a controlled inflammatory response after bleomycin-induced lung injury. Inflammation.

[34] Jiang G.X., Zhong X.Y., Cui Y.F., Liu W., Tai S., Wang Z.D., Shi Y.G., Zhao S.Y., Li C.L. (2010). IL-6/STAT3/TFF3 signaling regulates human biliary epithelial cell migration and wound healing *in vitro*. Mol. Biol. Rep.

[35] Mizoguchi A. (2012). Healing of intestinal inflammation by IL-22. Inflamm. Bowel. Dis.

[36] Nishida T., Nakamura M., Mishima H., Otori T. (1992). Interleukin 6 promotes epithelial migration by a fibronectin-dependent mechanism. J. Cell. Physiol.

[37] Pickert G., Neufert C., Leppkes M., Zheng Y., Wittkopf N., Warntjen M., Lehr H.A., Hirth S., Weigmann B., Wirtz S. (2009). STAT3 links IL-22 signaling in intestinal epithelial cells to mucosal wound healing. J. Exp. Med.

[38] Sugawara T., Gallucci R.M., Simeonova P.P., Luster M.I. (2001). Regulation and role of interleukin 6 in wounded human epithelial keratinocytes. Cytokine.

[39] Zenewicz L.A., Flavell R.A. (2011). Recent advances in IL-22 biology. Int. Immunol.

[40] Sallustio F., Costantino V., Cox S.N., Loverre A., Divella C., Rizzi M., Schena F.P. (2013). Human renal stem/progenitor cells repair tubular epithelial cell injury through TLR2-driven inhibin-A and microvesicle-shuttled decorin. Kidney Int.

[41] Brockes J.P. (1997). Amphibian limb regeneration: Rebuilding a complex structure. Science.

[42] Sipos F., Valcz G., Molnar B. (2012). Physiological and pathological role of local and immigrating colonic stem cells. World J. Gastroenterol.

[43] Ryu M., Migliorini A., Miosge N., Gross O., Shankland S., Brinkkoetter P.T., Hagmann H., Romagnani P., Liapis H., Anders H.J. (2012). Plasma leakage through glomerular basement membrane ruptures triggers the proliferation of parietal epithelial cells and crescent formation in non-inflammatory glomerular injury. J. Pathol.

[44] Yang L., Besschetnova T.Y., Brooks C.R., Shah J.V., Bonventre J.V. (2010). Epithelial cell cycle arrest in G2/M mediates kidney fibrosis after injury. Nat. Med.

[45] Liu Y. (2010). New insights into epithelial-mesenchymal transition in kidney fibrosis. J. Am. Soc. Nephrol.

[46] Kalluri R., Neilson E.G. (2003). Epithelial-mesenchymal transition and its implications for fibrosis. J. Clin. Invest.

[47] Niedermeier M., Reich B., Rodriguez Gomez M., Denzel A., Schmidbauer K., Gobel N., Talke Y., Schweda F., Mack M. (2009). CD4+ T cells control the differentiation of Gr1+ monocytes into fibrocytes. Proc. Natl. Acad. Sci. USA.

[48] Humphreys B.D., Lin S.L., Kobayashi A., Hudson T.E., Nowlin B.T., Bonventre J.V., Valerius M.T., McMahon A.P., Duffield J.S. (2010). Fate tracing reveals the pericyte and not epithelial origin of myofibroblasts in kidney fibrosis. Am. J. Pathol.

[49] Zeisberg M., Neilson E.G. (2010). Mechanisms of tubulointerstitial fibrosis. J. Am. Soc. Nephrol.

[50] Chapman K., Seldon M., Richards R. (2012). Thrombotic microangiopathies, thrombotic thrombocytopenic purpura, and ADAMTS-13. Semin. Thromb. Hemost.

[51] Amengual O., Atsumi T., Koike T. (2011). Pathophysiology of thrombosis and potential targeted therapies in antiphospholipid syndrome. Curr. Vasc. Pharmacol.

[52] Bonsib S.M. (1985). Glomerular basement membrane discontinuities. Scanning electron microscopic study of acellular glomeruli. Am. J. Pathol.

[53] Sorensen I., Susnik N., Inhester T., Degen J.L., Melk A., Haller H., Schmitt R. (2011). Fibrinogen, acting as a mitogen for tubulointerstitial fibroblasts, promotes renal fibrosis. Kidney Int.

[54] Drew A.F., Tucker H.L., Liu H., Witte D.P., Degen J.L., Tipping P.G. (2001). Crescentic glomerulonephritis is diminished in fibrinogen-deficient mice. Am. J. Physiol. Renal. Physiol.

[55] Downing L.J., Wakefield T.W., Strieter R.M., Prince M.R., Londy F.J., Fowlkes J.B., Hulin M.S., Kadell A.M., Wilke C.A., Brown S.L. (1997). Anti-P-selectin antibody decreases inflammation and thrombus formation in venous thrombosis. J. Vasc. Surg.

[56] Esmon C.T. (2005). The interactions between inflammation and coagulation. Br. J. Haematol.

[57] Loof T.G., Morgelin M., Johansson L., Oehmcke S., Olin A.I., Dickneite G., Norrby-Teglund A., Theopold U., Herwald H. (2011). Coagulation, an ancestral serine protease cascade, exerts a novel function in early immune defense. Blood.

[58] Loof T.G., Schmidt O., Herwald H., Theopold U. (2011). Coagulation systems of invertebrates and vertebrates and their roles in innate immunity: The same side of two coins?. J. Innate Immun.

[59] Rivers R.P., Hathaway W.E., Weston W.L. (1975). The endotoxin-induced coagulant activity of human monocytes. Br. J. Haematol.

[60] Cleary C.M., Moreno J.A., Fernández B., Ortiz A., Parra E.G., Gracia C., Blanco-Colio L.M., Barat A., Egido J. (2010). Glomerular haematuria, renal interstitial haemorrhage and acute kidney injury. Nephrol. Dial. Transpl.

[61] Degen J.L., Bugge T.H., Goguen J.D. (2007). Fibrin and fibrinolysis in infection and host defense. J. Thromb. Haemost.

[62] Nelson P.J., Rees A.J., Griffin M.D., Hughes J., Kurts C., Duffield J. (2012). The renal mononuclear phagocytic system. J. Am. Soc. Nephrol.

[63] Lech M., Avila-Ferrufino A., Skuginna V., Susanti H.E., Anders H.J. (2010). Quantitative expression of RIG-like helicase, NOD-like receptor and inflammasome-related mRNAs in humans and mice. Int. Immunol.

[64] Anders H.J., Banas B., Schlondorff D. (2004). Signaling danger: Toll-Like receptors and their potential roles in kidney disease. J. Am. Soc. Nephrol.

[65] Patole P.S., Pawar R.D., Lech M., Zecher D., Schmidt H., Segerer S., Ellwart A., Henger A., Kretzler M., Anders H.J. (2006). Expression and regulation of Toll-like receptors in lupus-like immune complex glomerulonephritis of MRL-Fas(lpr) mice. Nephrol. Dial. Transplant.

[66] Anders H.J., Schlondorff D. (2007). Toll-Like receptors: Emerging concepts in kidney disease. Curr. Opin. Nephrol. Hypertens.

[67] Anders H.J., Muruve D.A. (2011). The inflammasomes in kidney disease. J. Am. Soc. Nephrol.

[68] Pawar R.D., Castrezana-Lopez L., Allam R., Kulkarni O.P., Segerer S., Radomska E., Meyer T.N., Schwesinger C.M., Akis N., Grone H.J. (2009). Bacterial lipopeptide triggers massive albuminuria in murine lupus nephritis by activating Toll-like receptor 2 at the glomerular filtration barrier. Immunology.

[69] Patole P.S., Grone H.J., Segerer S., Ciubar R., Belemezova E., Henger A., Kretzler M., Schlondorff D., Anders H.J. (2005). Viral double-stranded RNA aggravates lupus nephritis through Toll-like receptor 3 on glomerular mesangial cells and antigen-presenting cells. J. Am. Soc. Nephrol.

[70] Anders H.J., Banas B., Linde Y., Weller L., Cohen C.D., Kretzler M., Martin S., Vielhauer V., Schlondorff D., Grone H.J. (2003). Bacterial CpG-DNA aggravates immune complex glomerulonephritis: Role of TLR9-mediated expression of chemokines and chemokine receptors. J. Am. Soc. Nephrol.

[71] Anders H.J., Vielhauer V., Eis V., Linde Y., Kretzler M., Perez de Lema G., Strutz F., Bauer S., Rutz M., Wagner H. (2004). Activation of toll-like receptor-9 induces progression of renal disease in MRL-Fas(lpr) mice. FASEB J.

[72] Allam R., Pawar R.D., Kulkarni O.P., Hornung V., Hartmann G., Segerer S., Akira S., Endres S., Anders H.J. (2008). Viral 5′-triphosphate RNA and non-CpG DNA aggravate autoimmunity and lupus nephritis via distinct TLR-independent immune responses. Eur. J. Immunol.

[73] Brown H.J., Lock H.R., Sacks S.H., Robson M.G. (2006). TLR2 stimulation of intrinsic renal cells in the induction of immune-mediated glomerulonephritis. J. Immunol.

[74] Brown H.J., Lock H.R., Wolfs T.G., Buurman W.A., Sacks S.H., Robson M.G. (2007). Toll-like receptor 4 ligation on intrinsic renal cells contributes to the induction of antibody-mediated glomerulonephritis via CXCL1 and CXCL2. J. Am. Soc. Nephrol.

[75] Brown H.J., Sacks S.H., Robson M.G. (2006). Toll-like receptor 2 agonists exacerbate accelerated nephrotoxic nephritis. J. Am. Soc. Nephrol.

[76] Wornle M., Schmid H., Banas B., Merkle M., Henger A., Roeder M., Blattner S., Bock E., Kretzler M., Grone H.J. (2006). Novel role of toll-like receptor 3 in hepatitis C-associated glomerulonephritis. Am. J. Pathol.

[77] Lichtnekert J., Vielhauer V., Zecher D., Kulkarni O.P., Clauss S., Segerer S., Hornung V., Mayadas T.N., Beutler B., Akira S. (2009). Trif is not required for immune complex glomerulonephritis: Dying cells activate mesangial cells via Tlr2/Myd88 rather than Tlr3/Trif. Am. J. Physiol. Renal. Physiol.

[78] Ryu M., Kulkarni O.P., Radomska E., Miosge N., Gross O., Anders H.J. (2011). Bacterial CpG-DNA accelerates Alport glomerulosclerosis by inducing an M1 macrophage phenotype and tumor necrosis factor-alpha-mediated podocyte loss. Kidney Int.

[79] Brahler S., Ising C., Hagmann H., Rasmus M., Hoehne M., Kurschat C., Kisner T., Goebel H., Shankland S., Addicks K. (2012). Intrinsic proinflammatory signaling in podocytes contributes to podocyte damage and prolonged proteinuria. Am. J. Physiol. Renal. Physiol.

[80] Lasagni L., Romagnani P. (2010). Glomerular epithelial stem cells: The good, the bad, and the ugly. J. Am. Soc. Nephrol.

[81] Pawar R.D., Patole P.S., Wornle M., Anders H.J. (2006). Microbial nucleic acids pay a Toll in kidney disease. Am. J. Physiol. Renal. Physiol.

[82] Marshak-Rothstein A., Rifkin I.R. (2007). Immunologically active autoantigens: The role of toll-like receptors in the development of chronic inflammatory disease. Annu. Rev. Immunol.

[83] Leadbetter E.A., Rifkin I.R., Hohlbaum A.M., Beaudette B.C., Shlomchik M.J., Marshak-Rothstein A. (2002). Chromatin-IgG complexes activate B cells by dual engagement of IgM and Toll-like receptors. Nature.

[84] Steinman R.M., Banchereau J. (2007). Taking dendritic cells into medicine. Nature.

[85] Migliorini A., Anders H.J. (2012). A novel pathogenetic concept-antiviral immunity in lupus nephritis. Nat. Rev. Nephrol.

[86] Anders H.J. (2009). Pseudoviral immunity—A novel concept for lupus. Trends Mol. Med.

[87] Anders H.J., Lichtnekert J., Allam R. (2010). Interferon-alpha and -beta in kidney inflammation. Kidney Int.

[88] Flur K., Allam R., Zecher D., Kulkarni O.P., Lichtnekert J., Schwarz M., Beutler B., Vielhauer V., Anders H.J. (2009). Viral RNA induces type I interferon-dependent cytokine release and cell death in mesangial cells via melanoma-differentiation-associated gene-5: Implications for viral infection-associated glomerulonephritis. Am. J. Pathol.

[89] Hagele H., Allam R., Pawar R.D., Anders H.J. (2009). Double-stranded RNA activates type I interferon secretion in glomerular endothelial cells via retinoic acid-inducible gene (RIG)-1. Nephrol. Dial. Transplant.

[90] Hagele H., Allam R., Pawar R.D., Reichel C.A., Krombach F., Anders H.J. (2009). Double-stranded DNA activates glomerular endothelial cells and enhances albumin permeability via a toll-like receptor-independent cytosolic DNA recognition pathway. Am. J. Pathol.

[91] Allam R., Lichtnekert J., Moll A., Taubitz A., Vielhauer V., Anders H.J. (2009). Viral RNA and DNA sense common antiviral responses including type I interferons in mesangial cells. J. Am. Soc. Nephrol.

[92] Fairhurst A.M., Mathian A., Connolly J.E., Wang A., Gray H.F., George T.A., Boudreaux C.D., Zhou X.J., Li Q.Z., Koutouzov S. (2008). Systemic IFN-alpha drives kidney nephritis in B6.Sle123 mice. Eur. J. Immunol.

[93] Fairhurst A.M., Xie C., Fu Y., Wang A., Boudreaux C., Zhou X.J., Cibotti R., Coyle A., Connolly J.E., Wakeland E.K. (2009). Type I interferons produced by resident renal cells may promote end-organ disease in autoantibody-mediated glomerulonephritis. J. Immunol.

[94] Pawar R.D., Ramanjaneyulu A., Kulkarni O.P., Lech M., Segerer S., Anders H.J. (2007). Inhibition of Toll-like receptor-7 (TLR-7) or TLR-7 plus TLR-9 attenuates glomerulonephritis and lung injury in experimental lupus. J. Am. Soc. Nephrol.

[95] Wu H., Ma J., Wang P., Corpuz T.M., Panchapakesan U., Wyburn K.R., Chadban S.J. (2010). HMGB1 contributes to kidney ischemia reperfusion injury. J. Am. Soc. Nephrol.

[96] Shigeoka A.A., Holscher T.D., King A.J., Hall F.W., Kiosses W.B., Tobias P.S., Mackman N., McKay D.B. (2007). TLR2 is constitutively expressed within the kidney and participates in ischemic renal injury through both MyD88-dependent and -independent pathways. J. Immunol.

[97] Leemans J.C., Stokman G., Claessen N., Rouschop K.M., Teske G.J., Kirschning C.J., Akira S., van der Poll T., Weening J.J., Florquin S. (2005). Renal-associated TLR2 mediates ischemia/reperfusion injury in the kidney. J. Clin. Invest.

[98] Wu H., Chen G., Wyburn K.R., Yin J., Bertolino P., Eris J.M., Alexander S.I., Sharland A.F., Chadban S.J. (2007). TLR4 activation mediates kidney ischemia/reperfusion injury. J. Clin. Invest.

[99] Allam R., Scherbaum C.R., Darisipudi M.N., Mulay S.R., Hagele H., Lichtnekert J., Hagemann J.H., Rupanagudi K.V., Ryu M., Schwarzenberger C. (2012). Histones from dying renal cells aggravate kidney injury via TLR2 and TLR4. J. Am. Soc. Nephrol.

[100] Saemann M.D., Weichhart T., Zeyda M., Staffler G., Schunn M., Stuhlmeier K.M., Sobanov Y., Stulnig T.M., Akira S., von Gabain A. (2005). Tamm-Horsfall glycoprotein links innate immune cell activation with adaptive immunity via a Toll-like receptor-4-dependent mechanism. J. Clin. Invest.

[101] Darisipudi M.N., Thomasova D., Mulay S.R., Brech D., Noessner E., Liapis H., Anders H.J. (2012). Uromodulin triggers IL-1beta-dependent innate immunity via the NLRP3 inflammasome. J. Am. Soc. Nephrol.

[102] Lech M., Garlanda C., Mantovani A., Kirschning C.J., Schlondorff D., Anders H.J. (2007). Different roles of TiR8/Sigirr on toll-like receptor signaling in intrarenal antigen-presenting cells and tubular epithelial cells. Kidney Int.

[103] Lassen S., Lech M., Rommele C., Mittruecker H.W., Mak T.W., Anders H.J. (2010). Ischemia reperfusion induces IFN regulatory factor 4 in renal dendritic cells, which suppresses postischemic inflammation and prevents acute renal failure. J. Immunol.

[104] Lech M., Avila-Ferrufino A., Allam R., Segerer S., Khandoga A., Krombach F., Garlanda C., Mantovani A., Anders H.J. (2009). Resident dendritic cells prevent postischemic acute renal failure by help of single Ig IL-1 receptor-related protein. J. Immunol.

[105] Gong J., Wei T., Stark R.W., Jamitzky F., Heckl W.M., Anders H.J., Lech M., Rossle S.C. (2010). Inhibition of Toll-like receptors TLR4 and 7 signaling pathways by SIGIRR: A computational approach. J. Struct. Biol.

[106] Banchereau J., Steinman R.M. (1998). Dendritic cells and the control of immunity. Nature.

[107] John R., Nelson P.J. (2007). Dendritic cells in the kidney. J. Am. Soc. Nephrol.

[108] Kruger T., Benke D., Eitner F., Lang A., Wirtz M., Hamilton-Williams E.E., Engel D., Giese B., Muller-Newen G., Floege J. (2004). Identification and functional characterization of dendritic cells in the healthy murine kidney and in experimental glomerulonephritis. J. Am. Soc. Nephrol.

[109] Vilaysane A., Chun J., Seamone M.E., Wang W., Chin R., Hirota S., Li Y., Clark S.A., Tschopp J., Trpkov K. (2010). The NLRP3 inflammasome promotes renal inflammation and contributes to CKD. J. Am. Soc. Nephrol.

[110] Lichtnekert J., Kulkarni O.P., Mulay S.R., Rupanagudi K.V., Ryu M., Allam R., Vielhauer V., Muruve D., Lindenmeyer M.T., Cohen C.D. (2011). Anti-GBM glomerulonephritis involves IL-1 but is independent of NLRP3/ASC inflammasome-mediated activation of caspase-1. PLoS One.

[111] Iyer S.S., Pulskens W.P., Sadler J.J., Butter L.M., Teske G.J., Ulland T.K., Eisenbarth S.C., Florquin S., Flavell R.A., Leemans J.C. (2009). Necrotic cells trigger a sterile inflammatory response through the Nlrp3 inflammasome. Proc. Natl. Acad. Sci. USA.

[112] Anders H.J., Vielhauer V., Schlondorff D. (2003). Chemokines and chemokine receptors are involved in the resolution or progression of renal disease. Kidney Int.

[113] Vielhauer V., Kulkarni O., Reichel C.A., Anders H.J. (2010). Targeting the recruitment of monocytes and macrophages in renal disease. Semin. Nephrol.

[114] Heller F., Lindenmeyer M.T., Cohen C.D., Brandt U., Draganovici D., Fischereder M., Kretzler M., Anders H.J., Sitter T., Mosberger I. (2007). The contribution of B cells to renal interstitial inflammation. Am. J. Pathol.

[115] Steinmetz O.M., Stahl R.A., Panzer U. (2009). Chemokines and B cells in renal inflammation and allograft rejection. Front. Biosci. (Schol Ed.).

[116] Swaminathan S., Griffin M.D. (2008). First responders: Understanding monocyte-lineage traffic in the acutely injured kidney. Kidney Int.

[117] Ley K., Laudanna C., Cybulsky M.I., Nourshargh S. (2007). Getting to the site of inflammation: The leukocyte adhesion cascade updated. Nat. Rev. Immunol.

[118] Stroo I., Stokman G., Teske G.J., Raven A., Butter L.M., Florquin S., Leemans J.C. (2010). Chemokine expression in renal ischemia/reperfusion injury is most profound during the reparative phase. Int. Immunol.

[119] Segerer S., Nelson P.J. (2005). Chemokines in renal diseases. ScientificWorldJournal.

[120] Ishida Y., Gao J.L., Murphy P.M. (2008). Chemokine receptor CX3CR1 mediates skin wound healing by promoting macrophage and fibroblast accumulation and function. J. Immunol.

[121] Panzer U., Kurts C. (2010). T cell cross-talk with kidney dendritic cells in glomerulonephritis. J. Mol. Med. (Berl. ).

[122] Anders H.J., Ryu M. (2011). Renal microenvironments and macrophage phenotypes determine progression or resolution of renal inflammation and fibrosis. Kidney Int.

[123] Lech M., Anders H.J. (2012). Macrophages and fibrosis: How resident and infiltrating mononuclear phagocytes orchestrate all phases of tissue injury and repair. Biochim. Biophys. Acta.

[124] Lech M., Grobmayr R., Weidenbusch M., Anders H.J. (2012). Tissues use resident dendritic cells and macrophages to maintain homeostasis and to regain homeostasis upon tissue injury: The immunoregulatory role of changing tissue environments. Mediators Inflamm.

[125] Duffield J.S. (2010). Macrophages and immunologic inflammation of the kidney. Semin. Nephrol.

[126] Anders H.J., Frink M., Linde Y., Banas B., Wornle M., Cohen C.D., Vielhauer V., Nelson P.J., Grone H.J., Schlondorff D. (2003). CC chemokine ligand 5/RANTES chemokine antagonists aggravate glomerulonephritis despite reduction of glomerular leukocyte infiltration. J. Immunol.

[127] Pawar R.D., Patole P.S., Ellwart A., Lech M., Segerer S., Schlondorff D., Anders H.J. (2006). Ligands to nucleic acid-specific toll-like receptors and the onset of lupus nephritis. J. Am. Soc. Nephrol.

[128] Anders H.J., Zecher D., Pawar R.D., Patole P.S. (2005). Molecular mechanisms of autoimmunity triggered by microbial infection. Arthritis Res. Ther.

[129] Ble A., Mosca M., Di Loreto G., Guglielmotti A., Biondi G., Bombardieri S., Remuzzi G., Ruggenenti P. (2011). Antiproteinuric effect of chemokine C-C motif ligand 2 inhibition in subjects with acute proliferative lupus nephritis. Am. J. Nephrol.

[130] Kulkarni O., Pawar R.D., Purschke W., Eulberg D., Selve N., Buchner K., Ninichuk V., Segerer S., Vielhauer V., Klussmann S. (2007). Spiegelmer inhibition of CCL2/MCP-1 ameliorates lupus nephritis in MRL-(Fas)lpr mice. J. Am. Soc. Nephrol.

[131] Ninichuk V., Clauss S., Kulkarni O., Schmid H., Segerer S., Radomska E., Eulberg D., Buchner K., Selve N., Klussmann S. (2008). Late onset of Ccl2 blockade with the Spiegelmer mNOX-E36–3′PEG prevents glomerulosclerosis and improves glomerular filtration rate in db/db mice. Am. J. Pathol.

[132] Kulkarni O., Eulberg D., Selve N., Zollner S., Allam R., Pawar R.D., Pfeiffer S., Segerer S., Klussmann S., Anders H.J. (2009). Anti-Ccl2 Spiegelmer permits 75% dose reduction of cyclophosphamide to control diffuse proliferative lupus nephritis and pneumonitis in MRL-Fas(lpr) mice. J. Pharmacol. Exp. Ther.

[133] Sayyed S.G., Ryu M., Kulkarni O.P., Schmid H., Lichtnekert J., Gruner S., Green L., Mattei P., Hartmann G., Anders H.J. (2011). An orally active chemokine receptor CCR2 antagonist prevents glomerulosclerosis and renal failure in type 2 diabetes. Kidney Int.

[134] Clauss S., Gross O., Kulkarni O., Avila-Ferrufino A., Radomska E., Segerer S., Eulberg D., Klussmann S., Anders H.J. (2009). Ccl2/Mcp-1 blockade reduces glomerular and interstitial macrophages but does not ameliorate renal pathology in collagen4A3-deficient mice with autosomal recessive Alport nephropathy. J. Pathol.

[135] Arnold L., Henry A., Poron F., Baba-Amer Y., van Rooijen N., Plonquet A., Gherardi R.K., Chazaud B. (2007). Inflammatory monocytes recruited after skeletal muscle injury switch into antiinflammatory macrophages to support myogenesis. J. Exp. Med.

[136] Gordon S., Taylor P.R. (2005). Monocyte and macrophage heterogeneity. Nat. Rev. Immunol.

[137] Lucas T., Waisman A., Ranjan R., Roes J., Krieg T., Muller W., Roers A., Eming S.A. (2010). Differential roles of macrophages in diverse phases of skin repair. J. Immunol.

[138] Xu J., Zhang X., Monestier M., Esmon N.L., Esmon C.T. (2011). Extracellular histones are mediators of death through TLR2 and TLR4 in mouse fatal liver injury. J. Immunol.

[139] Xu J., Zhang X., Pelayo R., Monestier M., Ammollo C.T., Semeraro F., Taylor F.B., Esmon N.L., Lupu F., Esmon C.T. (2009). Extracellular histones are major mediators of death in sepsis. Nat. Med.

[140] Kulkarni O.P., Ryu M., Kantner C., Sardy M., Naylor D., Lambert D., Brown R., Anders H.J. (2011). Recombinant chaperonin 10 suppresses cutaneous lupus and lupus nephritis in MRL-(Fas)lpr mice. Nephrol. Dial. Transplant.

[141] Vandewalle A. (2008). Toll-like receptors and renal bacterial infections. Chang. Gung Med. J.

[142] Ribeiro A., Wornle M., Motamedi N., Anders H.J., Grone E.F., Nitschko H., Kurktschiev P., Debiec H., Kretzler M., Cohen C.D. (2012). Activation of innate immune defense mechanisms contributes to polyomavirus BK-associated nephropathy. Kidney Int.

[143] Babel N., Volk H.D., Reinke P. (2011). BK polyomavirus infection and nephropathy: the virus-immune system interplay. Nat. Rev. Nephrol.

[144] Anders H.J., Patole P.S. (2005). Toll-like receptors recognize uropathogenic *Escherichia coli* and trigger inflammation in the urinary tract. Nephrol. Dial. Transplant.

[145] Patole P.S., Schubert S., Hildinger K., Khandoga S., Khandoga A., Segerer S., Henger A., Kretzler M., Werner M., Krombach F. (2005). Toll-like receptor-4: Renal cells and bone marrow cells signal for neutrophil recruitment during pyelonephritis. Kidney Int.

[146] Yang C.W., Hung C.C., Wu M.S., Tian Y.C., Chang C.T., Pan M.J., Vandewalle A. (2006). Toll-like receptor 2 mediates early inflammation by leptospiral outer membrane proteins in proximal tubule cells. Kidney Int.

[147] Bonventre J.V. (2003). Dedifferentiation and proliferation of surviving epithelial cells in acute renal failure. J. Am. Soc. Nephrol.

[148] Abuelo J.G. (2007). Normotensive ischemic acute renal failure. N. Engl. J. Med.

[149] Sugimoto H., Lebleu V.S., Bosukonda D., Keck P., Taduri G., Bechtel W., Okada H., Carlson W., Bey P., Rusckowski M. (2012). Activin-like kinase 3 is important for kidney regeneration and reversal of fibrosis. Nat. Med.

[150] Mulay S.R., Thomasova D., Ryu M., Anders H.J. (2012). MDM2 (murine double minute-2) links inflammation and tubular cell healing during acute kidney injury in mice. Kidney Int.

[151] Thomasova D., Mulay S.R., Bruns H., Anders H.J. (2012). p53-Independent Roles of MDM2 in NF-kappaB signaling: Implications for cancer therapy, wound healing, and autoimmune diseases. Neoplasia.

[152] Weidenbusch M., Anders H.J. (2012). Tissue microenvironments define and get reinforced by macrophage phenotypes in homeostasis or during inflammation, repair and fibrosis. J. Innate Immun.

[153] Ricardo S.D., van Goor H., Eddy A.A. (2008). Macrophage diversity in renal injury and repair. J. Clin. Invest.

[154] Duffield J.S., Park K.M., Hsiao L.L., Kelley V.R., Scadden D.T., Ichimura T., Bonventre J.V. (2005). Restoration of tubular epithelial cells during repair of the postischemic kidney occurs independently of bone marrow-derived stem cells. J. Clin. Invest.

[155] Lee S., Huen S., Nishio H., Nishio S., Lee H.K., Choi B.S., Ruhrberg C., Cantley L.G. (2011). Distinct macrophage phenotypes contribute to kidney injury and repair. J. Am. Soc. Nephrol.

[156] Zhang M.Z., Yao B., Yang S., Jiang L., Wang S., Fan X., Yin H., Wong K., Miyazawa T., Chen J. (2012). CSF-1 signaling mediates recovery from acute kidney injury. J. Clin. Invest.

[157] Duffield J.S., Forbes S.J., Constandinou C.M., Clay S., Partolina M., Vuthoori S., Wu S., Lang R., Iredale J.P. (2005). Selective depletion of macrophages reveals distinct, opposing roles during liver injury and repair. J. Clin. Invest.

[158] Lee S.B., Kalluri R. (2010). Mechanistic connection between inflammation and fibrosis. Kidney Int.

[159] Iwata Y., Bostrom E.A., Menke J., Rabacal W.A., Morel L., Wada T., Kelley V.R. (2012). Aberrant macrophages mediate defective kidney repair that triggers nephritis in lupus-susceptible mice. J. Immunol.

[160] Smeets B., Angelotti M.L., Rizzo P., Dijkman H., Lazzeri E., Mooren F., Ballerini L., Parente E., Sagrinati C., Mazzinghi B. (2009). Renal progenitor cells contribute to hyperplastic lesions of podocytopathies and crescentic glomerulonephritis. J. Am. Soc. Nephrol.

[161] Smeets B., Uhlig S., Fuss A., Mooren F., Wetzels J.F., Floege J., Moeller M.J. (2009). Tracing the origin of glomerular extracapillary lesions from parietal epithelial cells. J. Am. Soc. Nephrol.

[162] Atkins R.C., Nikolic-Paterson D.J., Song Q., Lan H.Y. (1996). Modulators of crescentic glomerulonephritis. J. Am. Soc. Nephrol.

[163] Tipping P.G., Kitching P.R., Holdsworth S.R., Neilson E.G., Couser W.G. (2001). The Formation of the Glomerular Crescent. Immunologic Renal Diseases.

[164] Bollee G., Flamant M., Schordan S., Fligny C., Rumpel E., Milon M., Schordan E., Sabaa N., Vandermeersch S., Galaup A. (2011). Epidermal growth factor receptor promotes glomerular injury and renal failure in rapidly progressive crescentic glomerulonephritis. Nat. Med.

[165] Ohse T., Pippin J.W., Chang A.M., Krofft R.D., Miner J.H., Vaughan M.R., Shankland S.J. (2009). The enigmatic parietal epithelial cell is finally getting noticed: A review. Kidney Int.

[166] Shankland S.J., Anders H.J., Romagnani P. (2013). Glomerular parietal epithelial cells in kidney physiology, pathology, and repair. Curr. Opin. Nephrol. Hypertens.

[167] Lindgren D., Bostrom A.K., Nilsson K., Hansson J., Sjolund J., Moller C., Jirstrom K., Nilsson E., Landberg G., Axelson H. (2011). Isolation and characterization of progenitor-like cells from human renal proximal tubules. Am. J. Pathol.

[168] Romagnani P. (2011). Family portrait: Renal progenitor of Bowman’s capsule and its tubular brothers. Am. J. Pathol.

[169] Forbes M.S., Thornhill B.A., Chevalier R.L. (2011). Proximal tubular injury and rapid formation of atubular glomeruli in mice with unilateral ureteral obstruction: A new look at an old model. Am. J. Physiol. Renal. Physiol.

[170] Chevalier R.L., Forbes M.S. (2008). Generation and evolution of atubular glomeruli in the progression of renal disorders. J. Am. Soc. Nephrol.

[171] D’Agati V.D., Kaskel F.J., Falk R.J. (2011). Focal segmental glomerulosclerosis. N. Engl. J. Med.

[172] Romagnani P., Remuzzi G. (2013). Renal progenitors in non-diabetic and diabetic nephropathies. Trends Endocrinol. Metab.

[173] Kriz W., Lemley K.V. (1999). The role of the podocyte in glomerulosclerosis. Curr. Opin. Nephrol. Hypertens.

[174] de Teixeira V.P., Blattner S.M., Li M., Anders H.J., Cohen C.D., Edenhofer I., Calvaresi N., Merkle M., Rastaldi M.P., Kretzler M. (2005). Functional consequences of integrin-linked kinase activation in podocyte damage. Kidney Int.

[175] Wharram B.L., Goyal M., Wiggins J.E., Sanden S.K., Hussain S., Filipiak W.E., Saunders T.L., Dysko R.C., Kohno K., Holzman L.B. (2005). Podocyte depletion causes glomerulosclerosis: Diphtheria toxin-induced podocyte depletion in rats expressing human diphtheria toxin receptor transgene. J. Am. Soc. Nephrol.

[176] Sachs N., Sonnenberg A. (2013). Cell-matrix adhesion of podocytes in physiology and disease. Nat. Rev. Nephrol.

[177] Lasagni L., Lazzeri E., Shankland S.J., Anders H.J., Romagnani P. (2013). Podocyte mitosis—A catastrophe. Curr. Mol. Med.

[178] Lasagni L., Ballerini L., Angelotti M.L., Parente E., Sagrinati C., Mazzinghi B., Peired A., Ronconi E., Becherucci F., Bani D. (2010). Notch activation differentially regulates renal progenitors proliferation and differentiation toward the podocyte lineage in glomerular disorders. Stem Cells.

[179] Pippin J.W., Durvasula R., Petermann A., Hiromura K., Couser W.G., Shankland S.J. (2003). DNA damage is a novel response to sublytic complement C5b-9-induced injury in podocytes. J. Clin. Invest.

[180] Mulay S.R., Thomasova D., Ryu M., Kulkarni O.P., Migliorini A., Bruns H., Gröbmayr R., Lazzeri E., Lasagni L., Liapis H., Romagnani P., Anders H.-J. (2013). Podocyte loss involves MDM2-driven mitotic catastrophe of podocytes. J. Pathol..

[181] Sugimoto H., Mundel T.M., Sund M., Xie L., Cosgrove D., Kalluri R. (2006). Bone-marrow-derived stem cells repair basement membrane collagen defects and reverse genetic kidney disease. Proc. Natl. Acad. Sci. USA.

[182] LeBleu V., Sugimoto H., Mundel T.M., Gerami-Naini B., Finan E., Miller C.A., Gattone V.H., Lu L., Shield C.F., Folkman J. (2009). Stem cell therapies benefit Alport syndrome. J. Am. Soc. Nephrol..

[183] Gross O., Borza D.B., Anders H.J., Licht C., Weber M., Segerer S., Torra R., Gubler M.C., Heidet L., Harvey S. (2009). Stem cell therapy for Alport syndrome: The hope beyond the hype. Nephrol. Dial. Transplant.

[184] Lazzeri E., Crescioli C., Ronconi E., Mazzinghi B., Sagrinati C., Netti G.S., Angelotti M.L., Parente E., Ballerini L., Cosmi L. (2007). Regenerative potential of embryonic renal multipotent progenitors in acute renal failure. J. Am. Soc. Nephrol.

[185] Ronconi E., Sagrinati C., Angelotti M.L., Lazzeri E., Mazzinghi B., Ballerini L., Parente E., Becherucci F., Gacci M., Carini M. (2009). Regeneration of glomerular podocytes by human renal progenitors. J. Am. Soc. Nephrol.

[186] Appel D., Kershaw D.B., Smeets B., Yuan G., Fuss A., Frye B., Elger M., Kriz W., Floege J., Moeller M.J. (2009). Recruitment of podocytes from glomerular parietal epithelial cells. J. Am. Soc. Nephrol.

[187] Sayyed S.G., Hagele H., Kulkarni O.P., Endlich K., Segerer S., Eulberg D., Klussmann S., Anders H.J. (2009). Podocytes produce homeostatic chemokine stromal cell-derived factor-1/CXCL12, which contributes to glomerulosclerosis, podocyte loss and albuminuria in a mouse model of type 2 diabetes. Diabetologia.

[188] Darisipudi M.N., Kulkarni O.P., Sayyed S.G., Ryu M., Migliorini A., Sagrinati C., Parente E., Vater A., Eulberg D., Klussmann S. (2011). Dual blockade of the homeostatic chemokine CXCL12 and the proinflammatory chemokine CCL2 has additive protective effects on diabetic kidney disease. Am. J. Pathol.

[189] Gaikwad A.B., Sayyed S.G., Lichtnekert J., Tikoo K., Anders H.J. (2010). Renal failure increases cardiac histone h3 acetylation, dimethylation, and phosphorylation and the induction of cardiomyopathy-related genes in type 2 diabetes. Am. J. Pathol.

[190] Sayyed S.G., Gaikwad A.B., Lichtnekert J., Kulkarni O., Eulberg D., Klussmann S., Tikoo K., Anders H.J. (2010). Progressive glomerulosclerosis in type 2 diabetes is associated with renal histone H3K9 and H3K23 acetylation, H3K4 dimethylation and phosphorylation at serine 10. Nephrol. Dial. Transplant.

[191] Angelotti M.L., Ronconi E., Ballerini L., Peired A., Mazzinghi B., Sagrinati C., Parente E., Gacci M., Carini M., Rotondi M. (2012). Characterization of renal progenitors committed toward tubular lineage and their regenerative potential in renal tubular injury. Stem Cells.

[192] Humphreys B.D., Czerniak S., DiRocco D.P., Hasnain W., Cheema R., Bonventre J.V. (2011). Repair of injured proximal tubule does not involve specialized progenitors. Proc. Natl. Acad. Sci. USA.

[193] Humphreys B.D., Valerius M.T., Kobayashi A., Mugford J.W., Soeung S., Duffield J.S., McMahon A.P., Bonventre J.V. (2008). Intrinsic epithelial cells repair the kidney after injury. Cell Stem Cell.

[194] Togel F.E., Westenfelder C. (2010). Mesenchymal stem cells: A new therapeutic tool for AKI. Nat. Rev. Nephrol.

[195] Higgins D.F., Lappin D.W., Kieran N.E., Anders H.J., Watson R.W., Strutz F., Schlondorff D., Haase V.H., Fitzpatrick J.M., Godson C. (2003). DNA oligonucleotide microarray technology identifies fisp-12 among other potential fibrogenic genes following murine unilateral ureteral obstruction (UUO): Modulation during epithelial-mesenchymal transition. Kidney Int.

[196] Ninichuk V., Gross O., Segerer S., Hoffmann R., Radomska E., Buchstaller A., Huss R., Akis N., Schlondorff D., Anders H.J. (2006). Multipotent mesenchymal stem cells reduce interstitial fibrosis but do not delay progression of chronic kidney disease in collagen4A3-deficient mice. Kidney Int.

[197] Migliorini A., Ebid R., Scherbaum C.R., Anders H.J. (2013). The danger control concept in kidney disease: mesangial cells. J. Nephrol.

[198] Johnson R.J., Raines E.W., Floege J., Yoshimura A., Pritzl P., Alpers C., Ross R. (1992). Inhibition of mesangial cell proliferation and matrix expansion in glomerulonephritis in the rat by antibody to platelet-derived growth factor. J. Exp. Med.

[199] Bomback A.S., Appel G.B. (2012). Pathogenesis of the C3 glomerulopathies and reclassification of MPGN. Nat. Rev. Nephrol.

[200] Hugo C., Shankland S.J., Bowen-Pope D.F., Couser W.G., Johnson R.J. (1997). Extraglomerular origin of the mesangial cell after injury. A new role of the juxtaglomerular apparatus. J. Clin. Invest.

[201] Imasawa T., Utsunomiya Y., Kawamura T., Zhong Y., Nagasawa R., Okabe M., Maruyama N., Hosoya T., Ohno T. (2001). The potential of bone marrow-derived cells to differentiate to glomerular mesangial cells. J. Am. Soc. Nephrol.

[202] Sethi S., Fervenza F.C. (2012). Membranoproliferative glomerulonephritis—A new look at an old entity. N. Engl. J. Med.

[203] Remuzzi G., Benigni A., Remuzzi A. (2006). Mechanisms of progression and regression of renal lesions of chronic nephropathies and diabetes. J. Clin. Invest.

[204] Smeets B., Kuppe C., Sicking E.M., Fuss A., Jirak P., van Kuppevelt T.H., Endlich K., Wetzels J.F., Grone H.J., Floege J. (2011). Parietal epithelial cells participate in the formation of sclerotic lesions in focal segmental glomerulosclerosis. J. Am. Soc. Nephrol.

[205] Helal I., Fick-Brosnahan G.M., Reed-Gitomer B., Schrier R.W. (2012). Glomerular hyperfiltration: Definitions, mechanisms and clinical implications. Nat. Rev. Nephrol.

[206] Bariety J., Hill G.S., Mandet C., Irinopoulou T., Jacquot C., Meyrier A., Bruneval P. (2003). Glomerular epithelial-mesenchymal transdifferentiation in pauci-immune crescentic glomerulonephritis. Nephrol. Dial. Transplant.

[207] Duffield J.S. (2010). Epithelial to mesenchymal transition in injury of solid organs: Fact or artifact?. Gastroenterology.

[208] Zeisberg M., Duffield J.S. (2010). Resolved: EMT produces fibroblasts in the kidney. J. Am. Soc. Nephrol.

[209] Kriz W., Kaissling B., Le Hir M. (2011). Epithelial-mesenchymal transition (EMT) in kidney fibrosis: Fact or fantasy?. J. Clin. Invest.

[210] Bohle A., Wehrmann M., Bogenschutz O., Batz C., Vogl W., Schmitt H., Muller C.A., Muller G.A. (1992). The long-term prognosis of the primary glomerulonephritides. A morphological and clinical analysis of 1747 cases. Pathol. Res. Pract.

[211] Famulski K.S., Reeve J., de Freitas D.G., Kreepala C., Chang J., Halloran P.F. (2013). Kidney transplants with progressing chronic diseases express high levels of acute kidney injury transcripts. Am. J. Transplant.

[212] Li Y., Liu Z., Guo X., Shu J., Chen Z., Li L. (2006). Aristolochic acid I-induced DNA damage and cell cycle arrest in renal tubular epithelial cells *in vitro*. Arch. Toxicol.

[213] Debelle F.D., Vanherweghem J.L., Nortier J.L. (2008). Aristolochic acid nephropathy: A worldwide problem. Kidney Int.

[214] Ninichuk V., Anders H.J. (2008). Bone marrow-derived progenitor cells and renal fibrosis. Front. Biosci.

[215] Vielhauer V., Anders H.J., Mack M., Cihak J., Strutz F., Stangassinger M., Luckow B., Grone H.J., Schlondorff D. (2001). Obstructive nephropathy in the mouse: Progressive fibrosis correlates with tubulointerstitial chemokine expression and accumulation of CC chemokine receptor 2- and 5-positive leukocytes. J. Am. Soc. Nephrol.

[216] Anders H.J., Vielhauer V., Kretzler M., Cohen C.D., Segerer S., Luckow B., Weller L., Grone H.J., Schlondorff D. (2001). Chemokine and chemokine receptor expression during initiation and resolution of immune complex glomerulonephritis. J. Am. Soc. Nephrol.

[217] Mayer V., Hudkins K.L., Heller F., Schmid H., Kretzler M., Brandt U., Anders H.J., Regele H., Nelson P.J., Alpers C.E. (2007). Expression of the chemokine receptor CCR1 in human renal allografts. Nephrol. Dial. Transplant.

[218] Vielhauer V., Anders H.J. (2009). Chemokines and chemokine receptors as therapeutic targets in chronic kidney disease. Front. Biosci. (Schol Ed. ).

[219] Jedlicka J., Soleiman A., Draganovici D., Mandelbaum J., Ziegler U., Regele H., Wuthrich R.P., Gross O., Anders H.J., Segerer S. (2010). Interstitial inflammation in Alport syndrome. Hum. Pathol.

[220] Anders H.J., Ninichuk V., Schlondorff D. (2006). Progression of kidney disease: Blocking leukocyte recruitment with chemokine receptor CCR1 antagonists. Kidney Int.

[221] Eis V., Vielhauer V., Anders H.J. (2004). Targeting the chemokine network in renal inflammation. Arch. Immunol. Ther. Exp. (Warsz).

[222] Eis V., Luckow B., Vielhauer V., Siveke J.T., Linde Y., Segerer S., Perez De Lema G., Cohen C.D., Kretzler M., Mack M. (2004). Chemokine receptor CCR1 but not CCR5 mediates leukocyte recruitment and subsequent renal fibrosis after unilateral ureteral obstruction. J. Am. Soc. Nephrol.

[223] Anders H.J., Vielhauer V., Frink M., Linde Y., Cohen C.D., Blattner S.M., Kretzler M., Strutz F., Mack M., Grone H.J. (2002). A chemokine receptor CCR-1 antagonist reduces renal fibrosis after unilateral ureter ligation. J. Clin. Invest.

[224] Anders H.J., Belemezova E., Eis V., Segerer S., Vielhauer V., Perez de Lema G., Kretzler M., Cohen C.D., Frink M., Horuk R. (2004). Late onset of treatment with a chemokine receptor CCR1 antagonist prevents progression of lupus nephritis in MRL-Fas(lpr) mice. J. Am. Soc. Nephrol.

[225] Vielhauer V., Berning E., Eis V., Kretzler M., Segerer S., Strutz F., Horuk R., Grone H.J., Schlondorff D., Anders H.J. (2004). CCR1 blockade reduces interstitial inflammation and fibrosis in mice with glomerulosclerosis and nephrotic syndrome. Kidney Int.

[226] Ninichuk V., Gross O., Reichel C., Khandoga A., Pawar R.D., Ciubar R., Segerer S., Belemezova E., Radomska E., Luckow B. (2005). Delayed chemokine receptor 1 blockade prolongs survival in collagen 4A3-deficient mice with Alport disease. J. Am. Soc. Nephrol.

[227] Ninichuk V., Khandoga A.G., Segerer S., Loetscher P., Schlapbach A., Revesz L., Feifel R., Khandoga A., Krombach F., Nelson P.J. (2007). The role of interstitial macrophages in nephropathy of type 2 diabetic db/db mice. Am. J. Pathol.

[228] Sakai N., Furuichi K., Shinozaki Y., Yamauchi H., Toyama T., Kitajima S., Okumura T., Kokubo S., Kobayashi M., Takasawa K. (2010). Fibrocytes are involved in the pathogenesis of human chronic kidney disease. Hum. Pathol.

[229] Wada T., Sakai N., Matsushima K., Kaneko S. (2007). Fibrocytes: A new insight into kidney fibrosis. Kidney Int.

[230] Reich B., Schmidbauer K., Rodriguez Gomez M., Johannes Hermann F., Gobel N., Bruhl H., Ketelsen I., Talke Y., Mack M. (2013). Fibrocytes develop outside the kidney but contribute to renal fibrosis in a mouse model. Kidney Int..

[231] Sakai N., Wada T., Yokoyama H., Lipp M., Ueha S., Matsushima K., Kaneko S. (2006). Secondary lymphoid tissue chemokine (SLC/CCL21)/CCR7 signaling regulates fibrocytes in renal fibrosis. Proc. Natl. Acad. Sci. USA.

[232] Duffield J.S., Lupher M., Thannickal V.J., Wynn T.A. (2013). Host responses in tissue repair and fibrosis. Annu. Rev. Pathol.

[233] Schrimpf C., Xin C., Campanholle G., Gill S.E., Stallcup W., Lin S.L., Davis G.E., Gharib S.A., Humphreys B.D., Duffield J.S. (2012). Pericyte TIMP3 and ADAMTS1 modulate vascular stability after kidney injury. J. Am. Soc. Nephrol.

[234] Fligny C., Duffield J.S. (2013). Activation of pericytes: Recent insights into kidney fibrosis and microvascular rarefaction. Curr. Opin. Rheumatol.

